# Evaluation of Pupal Parasitoids *Trichomalopsis ovigastra* and *Pachycrepoideus vindemiae* as Potential Biological Control Agents of *Bactrocera dorsalis*

**DOI:** 10.3390/insects16070708

**Published:** 2025-07-10

**Authors:** Ziwen Teng, Yiting Wang, Minghao Jiang, Yikun Zhang, Xintong Wang, Fanghao Wan, Hongxu Zhou

**Affiliations:** 1 Shandong Engineering Research Center for Environment-Friendly Agricultural Pest Management, Shandong Province Laboratory for Biological Invasions and Ecological Security, China-Australia Cooperative Research Center for Crop Health and Biological Invasions, College of Plant Health & Medicine, Qingdao Agricultural University, Qingdao 266109, China; tzwbat@126.com (Z.T.); 17863623108@163.com (Y.W.); jmhclear@163.com (M.J.); 13854270371@163.com (Y.Z.); 18654683937@163.com (X.W.); wanfanghao@caas.cn (F.W.); 2 Agricultural Genomics Institute at Shenzhen, Chinese Academy of Agricultural Sciences, Shenzhen 518000, China

**Keywords:** biological control, *Trichomalopsis ovigastra*, *Pachycrepoideus vindemiae*, *Drosophila melanogaster*, *Bactrocera dorsalis*

## Abstract

Parasitoid wasps are vital for biological control, and while new species continue to be discovered, evaluating their biological characteristics is crucial for realizing their potential for pest management. *Pachycrepoideus vindemiae* (Rondani) (Hymenoptera: Pteromalidae) is a well-studied parasitoid of dipteran pests, while *Trichomalopsis ovigastra* Sureshan & Narendran (Hymenoptera: Pteromalidae) has been only morphologically described. To assess its biocontrol potential, we compared the biological traits of *T. ovigastra* and *P. vindemiae* using *Drosophila melanogaster* Meigen (Diptera: Drosophilidae) and *Bactrocera dorsalis* (Hendel) (Diptera: Tephritidae) as hosts. *T. ovigastra* showed significantly higher parasitism rates, especially against *B. dorsalis*, where *T. ovigastra* achieved nearly 50% parasitism, compared to less than 0.3% by *P. vindemiae*. When using *D. melanogaster* as the host, no significant differences were observed between *T. ovigastra* and *P. vindemiae* in offspring sex ratio or adult longevity; however, *T. ovigastra* exhibited a shorter developmental duration and greater tolerance to temperature extremes, starvation, and desiccation. Notably, *B. dorsalis* has expanded its range from southern to northern China; however, no native parasitoids of this pest have been reported in the newly invaded northern regions. *T. ovigastra*, collected from northern orchards and capable of parasitizing *B. dorsalis*, thus shows promise as a biocontrol agent. These findings highlight the potential of locally occurring parasitoids, although field validation is still required.

## Simple Summary

Parasitoid wasps are well-known biocontrol agents, and discovering new species and examining their biological traits are essential to fully exploring their potential for pest management. In northern China, we collected *Trichomalopsis ovigastra* Sureshan & Narendran (Hymenoptera: Pteromalidae) directly from the field. This parasitoid species had previously only been described morphologically. In this study, we investigated additional biological characteristics of *T. ovigastra* and compared them with those of *Pachycrepoideus vindemiae* (Rondani) (Hymenoptera: Pteromalidae), a well-known parasitoid wasp of Diptera insects. Our results indicate that *T. ovigastra* exhibits stronger parasitism of both *Drosophila melanogaster* Meigen (Diptera: Drosophilidae) and *Bactrocera dorsalis* (Hendel) (Diptera: Tephritidae), shorter developmental times, and higher resistance to environmental stressors. Given the invasion of *B. dorsalis* into northern China and the absence of reported parasitoids in this region, our findings suggest that *T. ovigastra* could be a potential biocontrol agent for this invasive pest.

## 1. Introduction

The oriental fruit fly *Bactrocera dorsalis* (Hendel) (Diptera: Tephritidae) is a highly invasive pest species. Under natural field conditions, it has been recorded to have attacked 481 host taxa across 212 genera in 79 families, most notably Moraceae (38 taxa), Rutaceae (37 taxa), Solanaceae (33 taxa), and Cucurbitaceae (29 taxa) [1]. In China, *B. dorsalis* poses a severe threat to fruit production [2]. For example, in southern China, infestation rates on oranges reached 80–90% [3], and the national economic impacts on the citrus industry have been projected at approximately USD 40 billion [2]. Since its initial detection in Taiwan in 1912, *B. dorsalis* has steadily spread northward across mainland China [3]. Adults were first recorded on Hainan Island in the 1930s and have since colonized tropical and subtropical zones throughout the country [4]. More recently, *B. dorsalis* has been reported in temperate northern provinces such as Beijing, Hebei, and Henan [3,4,5]. Northern China, a major fruit-growing region producing peaches, apples, jujubes, and pears [6], now faces mounting risk; in Zhoukoudian (Beijing), plum infestation rates by *B. dorsalis* reached 10% [3]. To curb its further spread, China has implemented strict quarantine measures—such as trapping surveillance and postharvest thermal treatments—to detect and eliminate *B. dorsalis* infestations [7,8]. Although mass trapping (pheromone- and bait-based lures) [9], biological control (parasitoids, baculoviruses, and entomopathogenic fungi) [10,11,12], and the sterile insect technique [13] have been trialed, none have been widely adopted. Currently, synthetic insecticides remain the primary management tool, but their overuse has caused high-level resistance in *B. dorsalis* and has led to more severe outbreaks [14].

Parasitoid wasps play a pivotal role in structuring both natural and managed ecosystems [15] and hold substantial economic value as biocontrol agents against crop pests [16]. To harness their potential, comprehensive evaluations of candidate species’ traits—such as host range [17], parasitism efficacy [18], development and growth [19], stress tolerance [20], and host–parasitoid interactions [21]—are essential, because these parameters critically affect field performance [20,22,23,24].

Recent surveys of fruit fly parasitoids in orchards in northern China showed the occurrence of some resident parasitoid species attacking *B. dorsalis*. Among these species, *Trichomalopsis ovigastra* Sureshan & Narendran (Hymenoptera: Pteromalidae) and *Pachycrepoideus vindemiae* (Rondani) (Hymenoptera: Pteromalidae) were identified. *T. ovigastra* was first described from specimens collected in Kerala, India [25], but beyond that original record, its distribution and biology remain almost entirely undocumented. Our preliminary laboratory observations indicate that it can parasitize pupae of *Drosophila melanogaster* Meigen (Diptera: Drosophilidae) and of *B. dorsalis*. Although direct studies of *T. ovigastra* are scarce, research on other species within the genus *Trichomalopsis* may offer valuable insights. For example, Floate et al. have extensively studied *T. sarcophagae* (Gahan) for its host-searching behavior at different soil depths [26], comparative production on fresh versus freeze-killed hosts [27], cold tolerance [28], and biocontrol performance in cattle feedlots [29]. Other work on *Trichomalopsis* includes species descriptions [30], morphological and developmental analyses [31,32], parasitoid population surveys [33], host associations [34], and reproductive capacity assays [35]. Importantly, some *Trichomalopsis*—such as *T. apanteloctena* (Crawford)—act as facultative hyperparasitoids on hosts such as *Cotesia kariyai* (Watanabe) (Hymenoptera: Braconidae) [36] and *Apanteles* sp. (Hymenoptera, Braconidae) [37]. In contrast, *P. vindemiae* has been more extensively studied. *P. vindemiae* is a solitary, generalist pupal parasitoid first described in Italy [38] and now distributed in over 60 countries [39]. It has been reported to parasitize more than 60 fly species, including economically important species in the Tephritidae and Drosophilidae families [39,40,41,42]. Recent studies have focused on its morphology [43], parasitic behavior [42], field release [40], and the molecular mechanisms underlying host regulation [44]. It has shown significant potential as a biological control agent against dipteran pests [45] and has been evaluated primarily for controlling stable and house flies [46,47,48]. *P. vindemiae* can also act as a hyperparasitoid, necessitating caution when deploying it in systems involving other parasitoids [39].

*D. melanogaster*, which feeds on fermenting fruits, does not pose a significant agricultural threat. However, it serves as an excellent model organism in life sciences research [49]. Consequently, extensive research has been conducted on the mechanisms by which parasitoids regulate *D. melanogaster*, offering potential insights for applying parasitoids in biological control [50]. Using *D. melanogaster* as a host, our laboratory has previously studied the circadian activity, identified clock genes of *P. vindemiae* [42], and developed starvation-resistant lines via multi-generation artificial selection, assessing biological traits, such as starvation resistance, in both selected and non-selected lines [22]. In this study, we selected *D. melanogaster* as a secondary host model to compare the performance of *T. ovigastra* with that of the well-established ectoparasitoid *P. vindemiae*. Moreover, given the recent northward expansion of *B. dorsalis* into regions lacking native parasitoids, there is an urgent need to identify effective local natural enemies. Based on preliminary observations in our laboratory, we hypothesize that *T. ovigastra* may exhibit stronger parasitic ability against *B. dorsalis* than *P. vindemiae*, though experimental verification is still lacking. Therefore, this study had two objectives: (1) to compare *T. ovigastra* and *P. vindemiae* in key biocontrol traits—offspring production, sex ratio, developmental time, adult longevity, and resistance to temperature extremes, starvation, and desiccation—using *D. melanogaster* as a model host; (2) to assess *T. ovigastra*’s parasitism performance for *B. dorsalis* pupae, thereby evaluating its potential as a native biocontrol agent against this invasive pest.

## 2. Materials and Methods

### 2.1. Insect Rearing

The *D. melanogaster w^1118^* strain (Bloomington Drosophila Stock Center, Indiana University, Bloomington, IN, USA) was maintained on standard cornmeal-yeast medium. Colonies of *P. vindemiae* and *T. ovigastra* were established by parasitizing *D. melanogaster* pupae. *B. dorsalis* were sourced from South China Agricultural University and kept in an artificial climate room following the methods described by Zhu et al. [3]. We regularly monitored the survival rate, fecundity, and hatchability to prevent population degradation. Adults were housed in transparent culture tubes (2.5 × 10 cm) with 20% (*v*/*v*) sucrose solution. All insects were reared under controlled conditions at 25 °C, with 60 ± 5% relative humidity (RH) and a 12:12 L:D photoperiod.

### 2.2. Oviposition Behavior of T. ovigastra

Given the limited knowledge on *T. ovigastra* beyond morphological identification, preliminary observations of its oviposition behavior were conducted to confirm its parasitism of host pupae. A one-day-old, fully mated female wasp with no prior parasitism experience was individually introduced into a transparent Petri dish (8.5 cm diameter × 1.8 cm depth) containing a single two-day-old *D. melanogaster* pupa. The behavior was recorded under a high-resolution stereomicroscope (SangNond, Shenzhen, China) at 25 °C.

### 2.3. Offspring Number and Sex Ratio

In each culture tube (2.5 cm in diameter and 10 cm in height), 20 specific-age *D. melanogaster* pupae or 10 B. dorsalis pupae were introduced, along with a two-day-old, fully mated female wasp with no prior oviposition experience. The pupae ages for *D. melanogaster* were 0, 1, 2, and 3 days, with 0-day-old referring to pupae within 24 h of pupariation, 1-day-old referring to pupae that had been pupating for 1 day, and so on. For *B. dorsalis*, the pupae ages were 1, 3, 5, and 7 days. Following an initial 24 h parasitism period, fresh hosts were introduced daily. For *D. melanogaster*, we tested ten individual female wasps of each species and monitored their parasitism daily for 19 consecutive days (until each wasp reached 20 days of age). For *B. dorsalis*, we likewise tested ten individual female *T. ovigastra* and assessed parasitism over a 15-day period (until they reached 16 days of age). After the wasp offspring emerged, the number of males and females was recorded to calculate offspring production and sex ratio. In the initial experiments, we observed that *P. vindemiae* had a parasitism rate of less than 0.3% on *B. dorsalis* pupae, which was deemed inefficient and impractical for further analysis. As a result, *P. vindemiae* was excluded from the trials involving *B. dorsalis*.

### 2.4. Developmental Duration

The timing of oviposition was recorded, and adult emergence was monitored at 12 h intervals. The developmental time from egg to adult emergence was calculated separately for males and females. Twenty individuals per sex from each parasitoid species were assessed.

### 2.5. Longevity

Newly emerged, unmated male and female parasitoids were separated by sex, and each individual was placed into a separate tube. A 20% sucrose solution (Sangon Biotech, Shanghai, China) was provided as the nutritional source. Mortality was recorded every 12 h until all individuals had died. The assays were conducted at 25 °C and 60 ± 5% RH with a 12:12 h light:dark cycle.

### 2.6. High and Low Temperature Resistance

To assess thermal tolerance, unmated adults of defined ages (1, 15, and 30 days) from each parasitoid species were exposed individually to high (45 °C for 30 min) or low temperature (−10 °C for 2.5 h) conditions. Each sex was tested using 20 individuals. After thermal stress, the insects were returned to 25 °C, 60 ± 5% RH, and 12:12 h light:dark conditions. Survival was assessed 24 h post-treatment.

### 2.7. Starvation Resistance

Twenty newly emerged, unmated parasitoids from each species were individually maintained with access to water only. Mortality was recorded at 12 h intervals until all individuals died. The test conditions were 25 °C, 60 ± 5% RH, and a 12:12 h light:dark cycle.

### 2.8. Desiccation Resistance

Unmated wasps of both sexes and selected ages (1, 7, 15, 25, and 35 days) were tested individually. Twenty individuals of each sex per species were placed in separate tubes with 50 silica gel beads (3–5 mm) to induce desiccation. A sponge plug (1.2 cm height × 2.4 cm diameter) was placed above the beads to prevent direct contact with the insects. The tubes were sealed with Parafilm^®^ to reduce the relative humidity below 12% within 15 min. The wasps were held at 25 °C with a 12:12 h light:dark cycle, and mortality was recorded every 12 h.

### 2.9. Statistical Analysis

All statistical analyses were performed using R software (version 4.4.3) [51]. Offspring number and sex ratio were analyzed using generalized linear mixed models (GLMMs) with binomial distribution, considering parent ID as a random effect and parasitoid species as a fixed effect. The glmer function in the lme4 package (version 1.1.36) was used [52]. Visualizations were generated using ggplot2 (version 3.5.1). Cox regressions, implemented using the coxph function from the survival package (version 3.8.3) [53], were used to analyze developmental duration, lifespan, and survival under starvation and desiccation. To identify optimal covariates (species, sex, and interaction), models were compared based on Akaike Information Criterion (AIC), Bayesian Information Criterion (BIC), and likelihood ratio tests. The best-fitting model was selected using the lowest AIC/BIC and significant likelihood improvements. Survival curves were plotted with the ggsurvplot function from survminer (version 0.5.0) [54]. This study presents only the results of the best-fit model. Binary logistic regression using the glm function from stats (version 4.4.2) [51] was applied to analyze thermal resistance data. Model selection followed the same approach as above, evaluating AIC, BIC, and likelihood ratio tests to determine the most appropriate predictors (species, sex, and interaction).

## 3. Results

### 3.1. Oviposition Behavior of T. ovigastra

**Figure 1:**
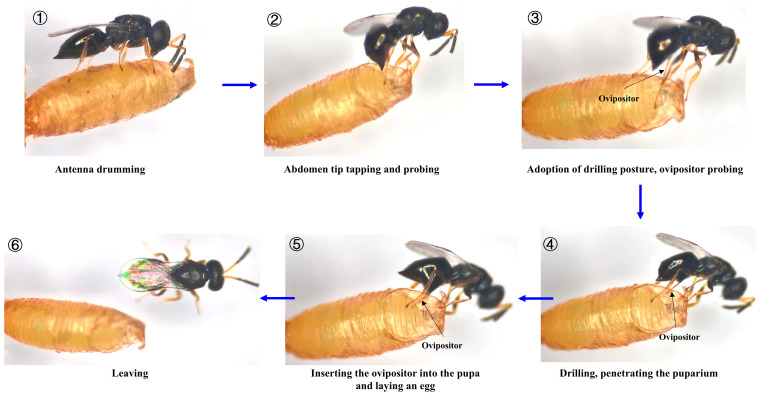
Sequence of the oviposition behavior of *Trichomalopsis ovigastra*.

The oviposition process of *T. ovigastra*, from antenna drumming to post-oviposition departure, was divided into six sequential phases: ① Antenna drumming: upon locating a host pupa, the female examined its surface by tapping it with her antennae. ② Abdomen tip tapping and probing: after antennal examination, the female curved her abdomen, gently probing the puparium surface with the tip. ③ Adoption of drilling posture and ovipositor probing: maintaining a curved posture, the female extended her ovipositor in preparation for penetration. ④ Drilling and penetrating the puparium: gradual rotational movements of the ovipositor characterized the initial drilling, transitioning to forceful thrusting or rocking motions to complete penetration. ⑤ Inserting the ovipositor into the pupa and laying an egg: the ovipositor was fully inserted into the host, where it was maneuvered internally for egg deposition. After laying the egg, the ovipositor was withdrawn. ⑥ Leaving: following oviposition, the female briefly touched the puparium with her antennae while walking over it, then moved away from the host (Figure 1).

### 3.2. Offspring Number and Sex Ratio

When parasitizing *D. melanogaster* pupae of different ages, *T. ovigastra* consistently produced more offspring than *P. vindemiae*. Specifically, each *T. ovigastra* female yielded mean offspring counts of 148.4, 234.8, 224.9, and 151.3 from 0-, 1-, 2-, and 3-day-old pupae, respectively, whereas each *P. vindemiae* female produced 79.1, 134.9, 138.9, and 84.7 offspring under the same conditions. Despite these differences in fecundity, the offspring sex ratio remained constant at 0.6 for both species across all pupal ages. For each parasitoid, the number of emerged offspring and sex ratios did not differ significantly across host pupal ages (Table 1).

To further evaluate the parasitism of the two species over time, we applied GLMMs with a binomial distribution, comparing offspring emergence across adult wasp ages. The results showed that from days 6, 8–11, and 13–20, *T. ovigastra* produced significantly more offspring than *P. vindemiae*, while no differences were found at other ages (Table 2 and Table S1).

**Figure 2:**
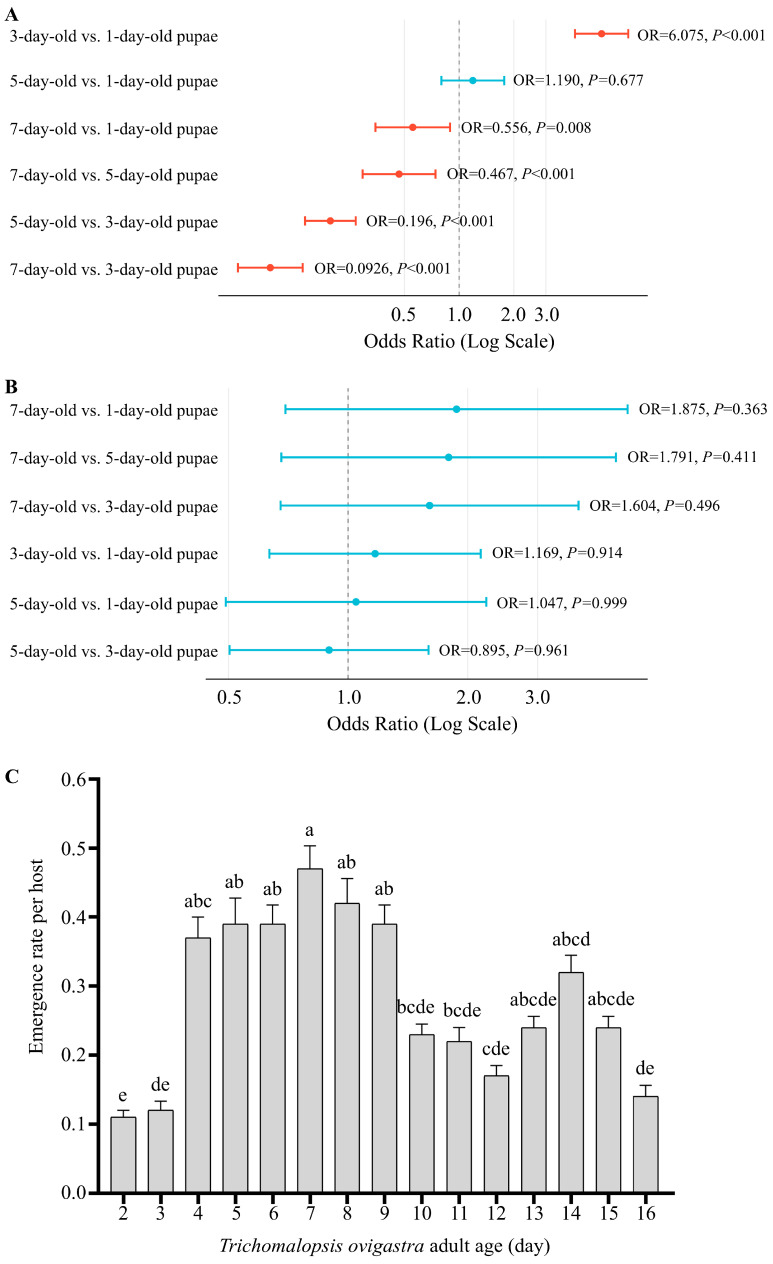
Offspring emergence and sex ratio of *Trichomalopsis ovigastra* parasitizing *Bactrocera dorsalis* pupae. (**A**) Number of emerged offspring from hosts of different pupal ages. (**B**) Sex ratio of parasitoid offspring emerging from hosts of different pupal ages. (**C**) Parasitism performance of *T. ovigastra* individuals of different ages on *B. dorsalis* pupae. Statistical analysis was performed using generalized linear mixed models with binomial distribution. In panel (**A**), red bars indicate pairwise comparisons with significant differences (*p* < 0.05). In panel (**C**), different lowercase letters above the bars denote statistically significant differences among groups (*p* < 0.05).

In the preliminary experiments, we found that *P. vindemiae* exhibited a parasitism rate below 0.3% on *B. dorsalis* pupae, making it unsuitable for subsequent analyses due to extremely low efficiency and lack of practical value. Consequently, we excluded *P. vindemiae* from the trials involving *B. dorsalis*. When *T. ovigastra* parasitized *B. dorsalis* pupae of different ages, significant differences in offspring emergence were observed. Except for the comparison between 5-day-old and 1-day-old pupae, where no significant differences were observed, all other age comparisons revealed significant differences. The highest emergence rate was recorded in 3-day-old pupae (Figure 2A and Table S2). However, the offspring sex ratio remained consistent across all pupal ages (Figure 2B and Table S2). In addition, parasitism by *T. ovigastra* differed significantly among individuals aged 2 to 16 days, with those at the younger and older ends of this age range exhibiting reduced parasitism efficiency (Figure 2C).

### 3.3. Developmental Duration and Longevity

**Figure 3:**
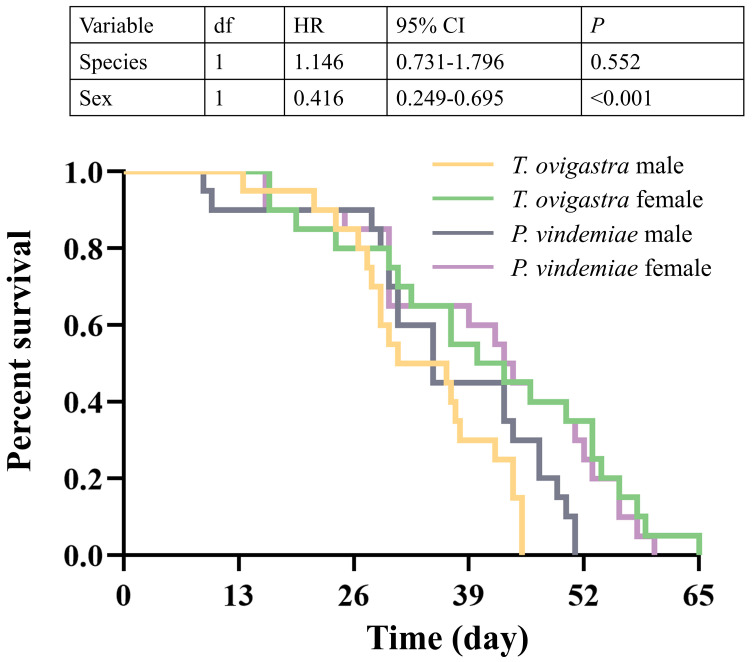
Comparison of longevity between two parasitoid species. Statistical analysis was conducted using Cox regression. *p* < 0.05 indicates a significant difference. HR: Hazard Ratio. CI: Confidence Interval.

*T. ovigastra* completed development from egg to adult emergence significantly faster than *P. vindemiae*. The females of both species exhibited prolonged developmental durations compared to the males (Table 3). No significant interspecific differences in adult longevity were detected between *T. ovigastra* and *P. vindemiae* (Figure 3).

### 3.4. High and Low Temperature Resistance

In the low-temperature resistance trials, no significant difference was found between the two parasitoids at day 1 post-emergence. However, at 15 and 30 days of age, *T. ovigastra* displayed significantly greater survival than *P. vindemiae*. In contrast, *T. ovigastra* consistently outperformed *P. vindemiae* at all the tested ages (1, 15, and 30 days) under high-temperature stress (Table 4).

### 3.5. Starvation Resistance

**Figure 4:**
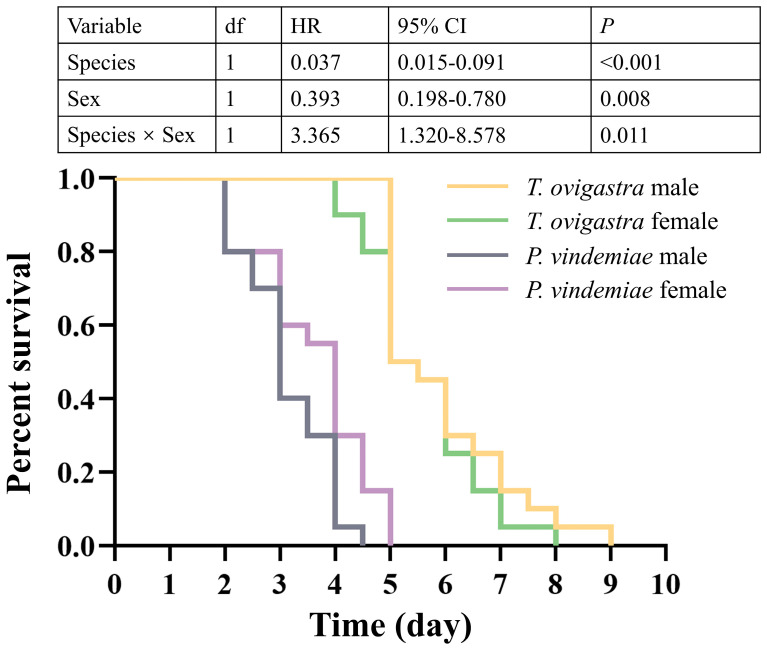
Comparison of starvation resistance between two parasitoid species. Statistical analysis was conducted using Cox regression. *p* < 0.05 indicates a significant difference. HR: hazard ratio. CI: confidence interval.

*T. ovigastra* exhibited significantly higher resistance to starvation than *P. vindemiae* (Figure 4). Moreover, no sex-related differences in starvation tolerance were observed in *T. ovigastra*, while in *P. vindemiae*, the females survived significantly longer than the males (Figure 4 and Table S3).

### 3.6. Desiccation Resistance

**Figure 5:**
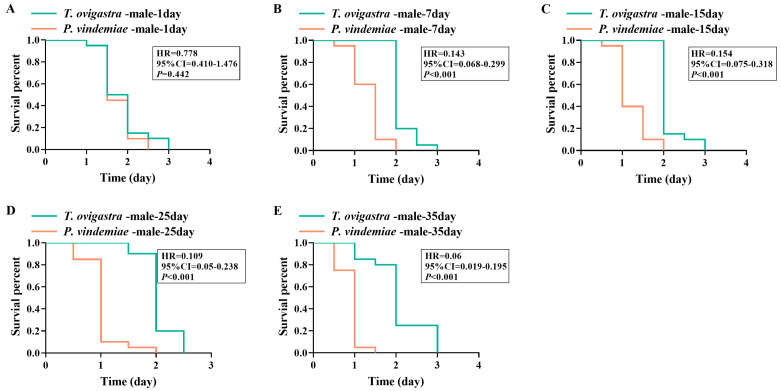
Desiccation resistance of male parasitoids from the two species. Survival rate of (**A**) 1-day-old males, (**B**) 7-day-old males, (**C**) 15-day-old males, (**D**) 25-day-old males, and (**E**) 35-day-old males under desiccation conditions. Statistical analysis was conducted using Cox regression. *p* < 0.05 indicates a significant difference. HR: hazard ratio. CI: confidence interval.

**Figure 6:**
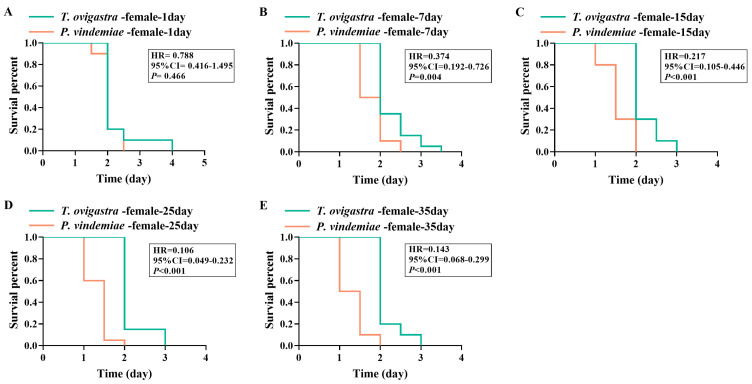
Desiccation resistance of female parasitoids from the two species. Survival rate of (**A**) 1-day-old females, (**B**) 7-day-old females, (**C**) 15-day-old females, (**D**) 25-day-old females, and (**E**) 35-day-old females under desiccation conditions. Statistical analysis was conducted using Cox regression. *p* < 0.05 indicates a significant difference. HR: hazard ratio. CI: confidence interval.

We assessed the desiccation tolerance across sexes and ages for both parasitoid species. On day 1, there was no difference in desiccation resistance between the two species’ male wasps (Figure 5A), nor between their females (Figure 6A). However, at all other tested ages, *T. ovigastra* exhibited significantly higher resistance than *P. vindemiae*, regardless of sex (Figure 5B–E and Figure 6B–E).

## 4. Discussion

Biological control constitutes a vital component of invasive pest management [55]. One strategy introduces and establishes natural enemies from the pest’s native range [56,57], but stringent regulations on importing non-native species often hinder its implementation [58]. An alternative and more straightforward approach enhances populations of resident natural enemies in newly invaded regions [58]. However, using parasitoids as an example, this strategy faces challenges as local parasitoids need varying amounts of time to adapt to new hosts, depending on their level of plasticity [59,60]. In our study, while *T. ovigastra* exhibited significantly higher parasitism than *P. vindemiae* on both hosts, its maximum emergence rate from *B. dorsalis* pupae remained below 50%. Such suboptimal parasitism likely reflects multiple ecological and physiological constraints, including incomplete host adaptation. Future studies should continue to measure *T. ovigastra*’s field parasitism rate on *B. dorsalis* to evaluate its host adaptation and pinpoint the factors limiting its efficacy.

Measuring offspring production over a limited interval provides an incomplete picture of a parasitoid’s reproductive potential compared to lifetime fecundity assessments [58]. For example, *P. vindemiae* maintains egg production throughout its adult life via both initial egg loads and ongoing oogenesis, whereas some species, such as *Trichopria drosophilae* Perkins (Hymenoptera: Diapriidae), cease oviposition midway through their lifespan due to arrested egg maturation [58,61]. Stacconi et al. (2017) suggested that these contrasting reproductive strategies may differentially influence the realized fecundity under field conditions, especially when the host availability fluctuates, potentially rendering *T. drosophilae* egg-limited in high-host environments [58]. Such insights could guide the selection of parasitoid species based on target pest densities. In this study, we counted offspring produced by parasitoids during the first 19 days on *D. melanogaster* and 15 days on *B. dorsalis*. Although *T. ovigastra* outperformed *P. vindemiae* between days 2 and 20, further studies are needed to determine the full duration and pattern of its oviposition period.

The number of offspring a parasitoid produces is strongly correlated with female longevity and, by extension, with adult nutritional intake [62,63,64]. Studies have demonstrated that *Ascogaster quadridentata* (Wesmael) (Hymenoptera: Braconidae), when provided with specific sugar or floral resources, experienced an extended lifespan. Moreover, the total number of parasitized host larvae per female was significantly correlated with the observed lifespan [62]. Hosts themselves can serve as important nutritional sources: host-feeding by *Eupelmus vuilletti* (Crawford) (Hymenoptera: Eupelmidae) boosts both egg production and survival [65], and female *P. vindemiae* that engage in host-feeding live significantly longer than those deprived of hosts [58]. It is hypothesized that females deprived of hosts may experience nutrient deficiencies, as they are unable to access the essential nutrients typically obtained through host-feeding [58]. We also observed host-feeding in *T. ovigastra*, but our longevity assays used only 20% sucrose solution. Future research should quantify survival when hosts are available.

The survival ability of parasitoids under unfavorable environmental conditions is also critical for their field biocontrol efficacy. For instance, releasing parasitoids at low pest densities is essential to prevent rapid growth of the target pest population, as delayed releases may hinder effective control. However, the absence of sufficient hosts can result in parasitoid extinction due to starvation. Thus, the starvation resistance of parasitoids is crucial for their survival and their ability to locate and parasitize hosts [66]. In this study, the laboratory data suggest that *T. ovigastra* may tolerate a wider range of temperatures and lower humidity levels compared to *P. vindemiae*. Consequently, these findings indicate that *T. ovigastra* may be more suitable for controlling *B. dorsalis*.

In conclusion, although this study provides valuable data on the parasitism, development, and stress tolerance of *T. ovigastra*, demonstrating its potential for biological control, several critical knowledge gaps remain. For instance, whether *T. ovigastra* exhibits facultative hyperparasitism—similar to *P. vindemiae*—is an urgent area for investigation. Notably, other species in the same genus, such as *T. apanteloctena*, are known to be idiobiont ecto-hyperparasitoids of prepupae or pupae of various *Cotesia* species [67]. Historical biocontrol programs reveal that hyperparasitism can undermine parasitoid efficacy [68]; for instance, *C. rubecula* (Marshall) experienced hyperparasitism rates rising from 37.9% in 1987 to 100% in August 1988, correlating with a failure to establish the agent in subsequent seasons [69]. Moreover, facultative hyperparasitoids that preferentially attack primary parasitoids over pest populations can be detrimental to control outcomes [70,71]. Therefore, determining whether *T. ovigastra* exhibits hyperparasitism and quantifying its host-selection preferences are essential steps. Additionally, research into its host-range breadth, diapause regulation, scalable rearing protocols, optimized release strategies, and molecular mechanisms of host manipulation will be indispensable for the safe and effective application of *T. ovigastra* in biocontrol programs.

## Figures and Tables

**Table 1:** Comparison of offspring emergence and sex ratio between two parasitoid species parasitizing *Drosophila melanogaster* pupae of different ages.

Response Variable	Explanatory Variable	Odds Ratio	95% Confidence Interval	*p*
Emerging parasitoid count	Intercept	0.353	0.287–0.434	<0.001
	Parasitoid species: *T. ovigastra* vs. *P. vindemiae*	2.494	2.020–3.080	<0.001
	Host pupa age (day)	1.014	0.923–1.115	0.763
Parasitoid sex ratio	Intercept	1.567	1.159–1.308	<0.001
	Parasitoid species: *T. ovigastra* vs. *P. vindemiae*	0.955	0.848–1.078	0.445
	Host pupa age (day)	1.012	0.957–1.069	0.673

**Table 2:** Offspring emergence of two parasitoid species parasitizing *Drosophila melanogaster* pupae at varying adult wasp ages.

Parasitoid Adult Age (Day)	Explanatory Variable	Odds Ratio	95% Confidence Interval	*p*
2	Intercept	0.709	0.534–0.938	0.017
	Parasitoid species: *T. ovigastra* vs. *P. vindemiae*	0.679	0.450–1.020	0.063
3	Intercept	1.740	1.309–2.330	<0.001
	Parasitoid species: *T. ovigastra* vs. *P. vindemiae*	0.899	0.599–1.347	0.606
4	Intercept	1.597	1.205–2.132	0.001
	Parasitoid species: *T. ovigastra* vs. *P. vindemiae*	0.797	0.534–1.187	0.264
5	Intercept	1.788	1.24–2.539	<0.001
	Parasitoid species: *T. ovigastra* vs. *P. vindemiae*	1.454	0.896–2.408	0.115
6	Intercept	1.817	1.356–2.483	<0.001
	Parasitoid species: *T. ovigastra* vs. *P. vindemiae*	2.687	1.687–4.408	<0.001
7	Intercept	1.597	1.205–2.131	0.001
	Parasitoid species: *T. ovigastra* vs. *P. vindemiae*	1.043	0.697–1.563	0.837
8	Intercept	0.681	0.511–0.901	0.008
	Parasitoid species: *T. ovigastra* vs. *P. vindemiae*	1.988	1.338–2.974	<0.001
9	Intercept	0.709	0.534–0.938	0.017
	Parasitoid species: *T. ovigastra* vs. *P. vindemiae*	1.794	1.209–2.673	0.004
10	Intercept	0.724	0.543–0.959	0.024
	Parasitoid species: *T. ovigastra* vs. *P. vindemiae*	1.907	1.282–2.865	0.001
11	Intercept	0.724	0.545–0.957	0.024
	Parasitoid species: *T. ovigastra* vs. *P. vindemiae*	1.907	1.284–2.844	0.001
12	Intercept	0.923	0.699–1.218	0.572
	Parasitoid species: *T. ovigastra* vs. *P. vindemiae*	1.222	0.825–1.811	0.318
13	Intercept	0.399	0.291–0.536	<0.001
	Parasitoid species: *T. ovigastra* vs. *P. vindemiae*	3.394	2.249–5.170	<0.001
14	Intercept	0.297	0.204–0.418	<0.001
	Parasitoid species: *T. ovigastra* vs. *P. vindemiae*	4.479	2.820–7.376	<0.001
15	Intercept	0.149	0.097–0.221	<0.001
	Parasitoid species: *T. ovigastra* vs. *P. vindemiae*	8.347	5.144–13.955	<0.001
16	Intercept	0.143	0.092–0.213	<0.001
	Parasitoid species: *T. ovigastra* vs. *P. vindemiae*	9.092	5.573–15.403	<0.001
17	Intercept	0.136	0.087–0.205	<0.001
	Parasitoid species: *T. ovigastra* vs. *P. vindemiae*	11.470	6.978–19.502	<0.001
18	Intercept	0.132	0.078–0.208	<0.001
	Parasitoid species: *T. ovigastra* vs. *P. vindemiae*	13.986	7.843–27.115	<0.001
19	Intercept	0.130	0.082–0.196	<0.001
	Parasitoid species: *T. ovigastra* vs. *P. vindemiae*	11.787	7.131–20.225	<0.001
20	Intercept	0.130	0.082–0.196	<0.001
	Parasitoid species: *T. ovigastra* vs. *P. vindemiae*	13.103	7.914–22.497	<0.001

**Table 3:** Cox regression analysis of developmental duration from egg to adult emergence in different parasitoid species.

Explanatory Variable	df	Hazard Ratio	95% Confidence Interval	*p*
Parasitoid species: *T. ovigastra* vs. *P. vindemiae*	1	265.363	33.117–345.399	<0.001
Sex: Female vs. Male	1	0.366	0.207–0.639	<0.001

Note: The reference categories are: *P. vindemiae* species and male sex.

**Table 4:** Logistic regression analysis of resistance to low and high temperatures in different parasitoid species.

Response Variable	Explanatory Variable	χ^2^	df	*p*	Odds Ratio
Low-temperature resistance in 1-day-old adult wasps ^1^	Parasitoid species: *T. ovigastra* vs. *P. vindemiae*	1.324	1	0.250	0.367
	Sex: Female vs. Male	0.158	1	0.691	1.378
Low-temperature resistance in 15-day-old adult wasps ^2^	Parasitoid species: *T. ovigastra* vs. *P. vindemiae*	12.811	1	<0.001	0.110
	Sex: Female vs. Male	0.293	1	0.588	0.744
Low-temperature resistance in 30-day-old adult wasps ^3^	Parasitoid species: *T. ovigastra* vs. *P. vindemiae*	16.499	1	<0.001	0.080
	Sex: Female vs. Male	0.668	1	0.414	0.637
High-temperature resistance in 1-day-old adult wasps ^4^	Parasitoid species: *T. ovigastra* vs. *P. vindemiae*	24.782	1	<0.001	0.018
	Sex: Female vs. Male	<0.001	1	1	1
High-temperature resistance in 15-day-old adult wasps ^5^	Parasitoid species: *T. ovigastra* vs. *P. vindemiae*	26.025	1	<0.001	0.003
	Sex: Female vs. Male	1.654	1	0.198	4.349
High-temperature resistance in 30-day-old adult wasps ^6^	Parasitoid species: *T. ovigastra* vs. *P. vindemiae*	27.519	1	<0.001	0.002
	Sex: Female vs. Male	0.956	1	0.328	3.162

Note: The reference categories are *P. vindemiae* species and male sex. An association is statistically significant if *p* is less than 0.050. ^1^: χ^2^ = 1.611, Nagelkerke *R*^2^ = 0.046, *p* = 0.447. ^2^: χ^2^ = 16.574, Nagelkerke *R*^2^ = 0.265, *p* < 0.001. ^3^: χ^2^ = 22.418, Nagelkerke *R*^2^ = 0.339, *p* < 0.001. ^4^: χ^2^ = 46.814, Nagelkerke *R*^2^ = 0.599, *p* < 0.001. ^5^: χ^2^ = 75.714, Nagelkerke *R*^2^ = 0.816, *p* < 0.001. ^6^: χ^2^ = 80.24, Nagelkerke *R*^2^ = 0.844, *p* < 0.001.

## Data Availability

The original contributions presented in this study are included in the article/Supplementary Materials. Further inquiries can be directed to the corresponding author.

## Supplementary Materials

The following supporting information can be downloaded at: https://www.mdpi.com/article/10.3390/insects16070708/s1. Table S1: Offspring emergence of two parasitoid species on *Drosophila melanogaster* pupae across adult wasp ages. Table S2: Offspring emergence and sex ratio of *Trichomalopsis ovigastra* on *Bactrocera dorsalis* pupae of varying ages. Table S3: Mixed effects Cox regression analysis of sex-specific differences in starvation resistance of parasitoid wasps.
