# Supplementary material for: Evaluation of Pupal Parasitoids Trichomalopsis ovigastra and Pachycrepoideus vindemiae as Potential Biological Control Agents of Bactrocera dorsalis
Source: Insects. 2025 Jul 10;16(7):708. doi: 10.3390/insects16070708 (PMC12295089; doi:10.3390/insects16070708)
Supplement: Supplementary file 1 [file insects-16-00708-s001.zip › Table S1.pdf]

Table S1 Offspring emergence of two parasitoid species on *Drosophila melanogaster* pupae across adult wasp ages

Wasp age (days)	*T. ovigastra* offspring emergence (mean ± SD)	*P. vindemiae* offspring emergence (mean ± SD)
2	6.4±2.2	8.3±1.6
3	12.2±2.0	12.7±1.6
4	11.2±2.3	12.3±1.5
5	14.4±2.8	12.8±1.8
6	16.6±2.3	12.9±1.5
7	12.5±2.1	12.3±1.4
8	11.5±2.0	8.1±2.2
9	11.2±1.9	8.3±1.5
10	11.6±2.5	8.4±1.9
11	11.6±1.9	8.4±2.0
12	10.6±2.3	9.6±1.6
13	11.5±1.8	5.7±0.9
14	11.4±3.1	4.6±1.0
15	11.1±2.2	2.6±1.2
16	11.3±2.2	2.5±1.4
17	12.2±2.1	2.4±1.1
18	12.9±3.0	2.4±1.2
19	12.1±2.3	2.3±0.8
20	12.6±2.2	2.3±0.9

The statistical data are detailed in Table 2.
