# Supplementary material for: Evaluation of Pupal Parasitoids Trichomalopsis ovigastra and Pachycrepoideus vindemiae as Potential Biological Control Agents of Bactrocera dorsalis
Source: Insects. 2025 Jul 10;16(7):708. doi: 10.3390/insects16070708 (PMC12295089; doi:10.3390/insects16070708)
Supplement: Supplementary file 1 [file insects-16-00708-s001.zip › Table S2.pdf]

Table S2 Offspring emergence and sex ratio of *Trichomalopsis ovigastra* on *Bactrocera dorsalis* pupae of varying ages

Host pupal age (days)	Offspring emergence (mean ± SD)	Offspring sex ratio (mean ± SD)
1	9.1±2.8	0.6±0.2
3	42.2±7.5	0.7±0.0
5	10.7±3.2	0.6±0.1
7	5.2±1.9	0.8±0.2

The statistical data are detailed in Figure 2A and 2B.
