# Supplementary material for: Evaluation of Pupal Parasitoids Trichomalopsis ovigastra and Pachycrepoideus vindemiae as Potential Biological Control Agents of Bactrocera dorsalis
Source: Insects. 2025 Jul 10;16(7):708. doi: 10.3390/insects16070708 (PMC12295089; doi:10.3390/insects16070708)
Supplement: Supplementary file 1 [file insects-16-00708-s001.zip › Table S3.pdf]

**Table S3** Mixed effects Cox regression analysis of sex-specific differences in starvation resistance of parasitoid wasps

| Dependent Variable | Variable | df | Hazard Ratio | 95% Confidence Interval | *p* |
|---|---|---|---|---|---|
| *T. ovigastra* survival under starvation condition | Female vs. Male | 1 | 1.335 | 0.708-2.517 | 0.372 |
| *P. vindemiae* survival under starvation condition | Female vs. Male | 1 | 0.480 | 0.244-0.946 | 0.034 |

Note: For the Cox regression model, the reference categories are: male sex.
